# Altered Lipid and Salt Taste Responsivity in Ghrelin and GOAT Null Mice

**DOI:** 10.1371/journal.pone.0076553

**Published:** 2013-10-04

**Authors:** Huan Cai, Wei-na Cong, Caitlin M. Daimon, Rui Wang, Matthias H. Tschöp, Jean Sévigny, Bronwen Martin, Stuart Maudsley

**Affiliations:** 1 Metabolism Unit, Laboratory of Clinical Investigation, National Institute on Aging, National Institutes of Health, Baltimore, Maryland, United States of America; 2 Institute for Diabetes and Obesity, Helmholtz Centre Munich, Munich, Germany; 3 Centre de recherche en Rhumatologie et Immunologie, Centre de recherche du CHU de Québec, QC and Département de microbiologie-infectiologie et d′immunologie, Faculté de médecine, Université Laval, Québec, Quebec City, Canada; 4 Receptor Pharmacology Unit, Laboratory of Neuroscience, National Institute on Aging, National Institutes of Health, Baltimore, Maryland, United States of America; German Institute for Human Nutrition, Germany

## Abstract

Taste perception plays an important role in regulating food preference, eating behavior and energy homeostasis. Taste perception is modulated by a variety of factors, including gastric hormones such as ghrelin. Ghrelin can regulate growth hormone release, food intake, adiposity, and energy metabolism. Octanoylation of ghrelin by ghrelin O-acyltransferase (GOAT) is a specific post-translational modification which is essential for many biological activities of ghrelin. Ghrelin and GOAT are both widely expressed in many organs including the gustatory system. In the current study, overall metabolic profiles were assessed in wild-type (WT), ghrelin knockout (ghrelin^−/−^), and GOAT knockout (GOAT^−/−^) mice. Ghrelin^−/−^ mice exhibited decreased food intake, increased plasma triglycerides and increased ketone bodies compared to WT mice while demonstrating WT-like body weight, fat composition and glucose control. In contrast GOAT^−/−^ mice exhibited reduced body weight, adiposity, resting glucose and insulin levels compared to WT mice. Brief access taste behavioral tests were performed to determine taste responsivity in WT, ghrelin^−/−^ and GOAT^−/−^ mice. Ghrelin and GOAT null mice possessed reduced lipid taste responsivity. Furthermore, we found that salty taste responsivity was attenuated in ghrelin^−/−^ mice, yet potentiated in GOAT^−/−^ mice compared to WT mice. Expression of the potential lipid taste regulators Cd36 and Gpr120 were reduced in the taste buds of ghrelin and GOAT null mice, while the salt-sensitive ENaC subunit was increased in GOAT^−/−^ mice compared with WT mice. The altered expression of Cd36, Gpr120 and ENaC may be responsible for the altered lipid and salt taste perception in ghrelin^−/−^ and GOAT^−/−^ mice. The data presented in the current study potentially implicates ghrelin signaling activity in the modulation of both lipid and salt taste modalities.

## Introduction

Gustation is one of the fundamental chemical senses that guides organisms to identify nutrients while avoiding toxic chemicals. This sensory mechanism is primarily mediated through ion channels or receptors in taste cells which are clustered into onion-shaped taste buds. Taste buds are located within three different types of papillae: fungiform papillae located in the apical region of the tongue, foliate papillae on the lateral posterior tongue and circumvallate papillae on the posterior tongue [1]. Differences in ultrastructural features, stage of differentiation and diverse functioning allows taste cells within the taste buds to be classified into Type I, II, III and IV taste cells. Type I taste cells, the most abundant cells in the tongue, function as supportive glia in taste buds [2]. Type II cells are commonly referred to as ‘receptor cells’ as they express a variety of sweet, bitter and umami taste receptors [3]. After tastant binding to a specific taste receptor, taste cells are activated and signals are transmitted to sensory afferents or other cells within the taste bud [4]. Type III cells, known as ‘presynaptic cells’, express multiple synaptic proteins and demonstrate functional depolarization-dependent Ca^2+^ transients [5], [6]. Type IV taste cells are non-polarized, undifferentiated basal cells that are thought to represent a latent stem cell-like population in the tongue [7].

Typically, tastant molecules are recognized as being associated with one of five basic taste modalities: sweet, sour, bitter, salty, and umami. The sweet taste modality signals the presence of carbohydrate energy sources, while the aversive taste modalities, sour and bitter, can help the organism defend against acids or poisons. Salty taste governs the intake of Na^+^ which is essential for maintaining the body's osmotic balance and cardiovascular fluid volume. Low concentration of sodium is considered an appetitive stimulus, while higher concentration generates an aversive gustatory response. Recent studies have demonstrated that the epithelial sodium channel (ENaC) is expressed in taste cells and is primarily responsible for the detection of salty taste in mice [8], [9], [10]. There is increasing evidence suggesting the existence of a sixth taste modality, known as the lipid taste [11]. The perception of lipids is partially dependent on the texture of the tastant, but mostly determined by oral detection of dietary fats. Although dietary lipids consist mainly of triglycerides, free fatty acids other than triglycerides are also effective taste stimuli in animal models [12], [13].

Acyl ghrelin (AG), a 28-amino acid peptide, was first identified as the endogenous ligand for the growth hormone secretagogue receptor (GHS-R1a) from rat stomach by Kojima et al. [14]. Des-acyl ghrelin (DAG), des-acyl des-Gln^14^-ghrelin and obestatin are three protein products of the ghrelin gene [15]. The 94-amino acid pro-ghrelin is generated by removing the signal sequence of pre-pro-ghrelin. After cleavage of pro-ghrelin by prohormone convertase PC1/3, the N-terminal fragments generate des-acyl ghrelin and des-acyl des-Gln^14^-ghrelin, whereas obestatin is derived from the C-terminal fragments [16]. O-n-octanoylation at serine 3 is a unique post-translational modification (PTM) required for acyl ghrelin to bind to its receptor GHS-R1a to stimulate growth hormone secretion. The lipid transferase, ghrelin O-acyltransferase (GOAT), which attaches octanoate to serine-3 of ghrelin, was independently identified by two research groups [17], [18]. GOAT belongs to the membrane-bound O-acyltransferases (MBOAT) superfamily and is a porcupine-like enzyme. GOAT expression consistently overlaps with that of ghrelin in most endocrine organ systems such as the gastrointestinal tract, hypothalamus-pituitary axis, pancreas, reproductive system, and the bone and gustatory systems [19]. This functional ghrelin/GOAT system plays a role in controlling energy, lipid, and glucose metabolism [20], [21], hence acylated ghrelin administration leads to increased food intake, fat accumulation and body weight [22], [23], [24], [25]. Interestingly, the expression of ghrelin and GOAT and the activation of the ghrelin/GOAT system is modified by dietary lipids, especially medium-chain fatty acids [16], [26]. Des-acyl ghrelin (DAG) has also been demonstrated to be a functionally important peptide ligand as well *i.e.* DAG can act as a functional antagonist of AG signaling [27]. DAG also appears to possess some AG-independent activity as well. Obese metabolic syndrome patients can exhibit lower DAG but comparable AG and higher AG/DAG ratios [28], however a low AG/DAG ratio is associated with an improved metabolic state [29].

Not only can ghrelin control food intake, it also has effects on macronutrient selection, *e.g*. central administration of ghrelin preferentially enhanced fat consumption over carbohydrates in both high-fat and high-carbohydrate-preferring rats [30]. A recent study in our laboratory has also demonstrated that ghrelin, GOAT, GHSR, prepro-ghrelin and prohormone convertase 1/3 (PC 1/3) are expressed in Type I, II, III and IV taste cells of mouse taste buds, indicating that they could play local modulatory roles in taste perception [31]. In our study we found that GOAT and ghrelin co-localize in many taste cells, however ghrelin immunopositive cells were often observed that did not apparently express significant levels of GOAT. Ghrelin and its receptors have also been shown to be present in human salivary glands and ghrelin is secreted into saliva [32]. Despite such emerging information, no study to date has investigated the gustatory perceptive system in both ghrelin and GOAT null mice. This comparative question is important given the presence of GOAT positive and negative ghrelin-expressing cells in the tongue as well as the differential pharmacological activities of AG and DAG. In the present study, we aimed to assess the overall metabolic profiles of ghrelin^−/−^ and GOAT^−/−^ mice, in addition to investigating whether ghrelin and GOAT null mice display differential responses to specific taste modalities. Our data indicate that although the morphology and qualitative nature of taste cells have not been altered in ghrelin and GOAT knockout mice, the expression of some salty and lipid taste related modulatory proteins has been changed in ghrelin^−/−^ and GOAT^−/−^ mice. Our current data demonstrate that the ghrelin signaling system is an important regulator of lipid and salt sensation and therefore may present itself as a future therapeutic target in which lipid or salt ingestion and metabolism can be modified to prevent pathophysiological states such as diet-induced obesity or hypertension.

## Materials and Methods

### Animals and Tissue Collection

All animal procedures were approved by the Animal Care and Use Committee of the National Institute on Aging, National Institutes of Health in Baltimore, MD (NIA protocol number: 397-LCI-2012). Male ghrelin^−/−^ and GOAT^−/−^ mice on a BL6/C57 background and their wild-type counterparts were employed for our studies. Ghrelin and GOAT null mice were provided by Dr. Matthias Tschöp. The body weight was measured at the end of the study. The body composition was analyzed by dual-energy X-ray absorptiometry (DEXA) (Lunar, PIXImus, Fitchburg, WI). Upon completion of behavior analyses (described below), animals were anesthetized using Isoflurane (Butler Animal Health Supply, Vancouver, WA) and immediately decapitated upon verification of unconsciousness. Trunk blood was immediately collected following decapitation and centrifuged at 3000 rpm for 30 minutes at 4°C. Plasma was subsequently collected. Tongues were carefully collected from each animal as described previously [33], [34]. Excised tongues were fixed in 4% paraformaldehyde (Sigma, St. Louis, MO) for 1 hour and then cryoprotected with 20% sucrose in 0.1 M phosphate buffer overnight at 4°C. Serial sections (8–10 µm thickness) were cut from the tissues containing fungiform, foliate and circumvallate papillae using a cryostat (HM 500M, MICRON, Laborgerate GmbH, Germany). All experimental mice were fasted overnight before sacrifice.

### Behavioral taste testing

Behavioral assessment of taste responsivity was performed as previously described [33], [34], [35]. All taste testing took place during daylight hours, and all mice were habituated to the laboratory environment for 30 minutes each day prior to the initiation of taste testing. All tastants were prepared with purified water from the NIA animal facility and reagent-grade chemicals were presented to the animals at room temperature (21–22°C). Test stimuli consisted of various concentrations of sucrose (25, 75, 150, 300, 600 and 1000 mM; Fisher Scientific, Atlanta, GA), sodium chloride (NaCl:15, 100, 300, 500, 600 and 1000 mM; Sigma), denatonium benzoate (DB: 0.001, 0.01, 0.1, 0.3, 1 and 3 mM; Sigma), citric acid (CA: 0.1, 0.5, 1, 3, 5, 10, 20, 30, and 100 mM; Fisher Scientific), and intralipid (0.1%, 1%, 5%, 10%, and 20%: Sigma). Brief-access taste testing took place in a Davis MS-160 gustometer (DiLog Instruments, Tallahassee, FL) as previously described [35]. Brief-access procedures minimize post-ingestive effects that may confound other assays such as intake tests or 2-bottle taste tests. Mice accessed the taste stimuli (presented as an ascending concentration range) or water in sipper bottles through a small opening in the mouse chamber. Before taste testing was initiated, mice were trained to lick a stationary tube of water in the gustometer after being placed on a 23.5 hour restricted water-access schedule. Unconditioned licking responses were recorded for later analyses in 25 minute brief-access test sessions, during which mice could initiate as many trials as possible in this period. Stimulus presentation order was randomized within blocks. The duration of each trial (5 seconds) was regulated by a computer-controlled shutter that allowed access to the sipper tube. There was a 7.5 second inter-presentation interval, during which time a stepper motor moved one of up to seven tubes (containing water or a specific concentration of tastant) in front of the shuttered opening. Two different testing protocols were used: one for appetitive stimuli (sucrose, intralipid) and one for aversive stimuli (NaCl, DB, CA). For sucrose and intralipid, animals received 5 days of testing using the various stimulus concentrations and purified NIA animal facility water. Prior to each day of sucrose and intralipid testing, animals were placed on a 23.5 hour restricted food and water-access schedule (1 gram of food and 2 mL of water) in order to maintain motivation to drink, and thus increasing the number of stimulus presentations taken during testing. In a similar manner for NaCl, CA, and DB, animals received 5 days of testing with the five stimulus concentrations and with purified NIA animal facility water. Similarly to the testing performed with sucrose and intralipid, the mice were water-restricted during NaCl, DB, and CA testing in order to increase the number of stimulus presentations taken. Additionally, a water rinse presentation (1 s) was interposed between the test trials for NaCl, DB, and CA to help control for any potential tastant carry-over effects. During the behavioral experiments, the animals were equally exposed to water and a serial concentration of tastants. Finally, the average number of licks per trial for each stimulus concentration was divided by the average number of water licks per trial, yielding a tastant/water lick ratio. This ratio controls for individual differences in motivational state. Additionally, a 2-bottle taste preference test was carried out as described previously [36]. Preference was characterized by calculating the ratio of intralipid (15%) lick number to water lick number over 48 h.

### Data analysis and statistical methods for behavioral taste testing

The average number of licks per trial for each stimulus concentration was divided by the average number of water licks per trial, yielding a tastant/water lick ratio. This ratio controls for individual differences in motivational state. The ratios were analyzed with standard ANOVA using GraphPad Prism (v5.0). The conventional *p*≤0.05 was applied as the statistical rejection criterion. Tastant concentration-lick ratio response curves were fitted to the mean data for each group using a classical four parameter logistic sigmoidal dose-response equation using the non-linear regression suite of GraphPad Prism (v5.0).

### Whole-animal metabolic behavioral analysis

A comprehensive lab animal metabolic monitoring system (CLAMS; Columbus Instruments, Columbus, OH) was employed to collect data concerning mouse ambulatory activity (x, z axis motion counts), energy expenditure (kL/hr), O_2_ consumption (mL/Kg/hr), CO_2_ production (mL/Kg/hr) and respiratory exchange ratio (RER  =  Vco_2_/Vo_2_), as described previously [37], [38]. Energy expenditure was calculated from the gas exchange data [energy expenditure  =  (3.815+1.232 ×RER) ×V_O2_] and expressed as kJ/kg/h. RER is strongly controlled by the physico-chemical nature of the primary energy source the animal is using, *i.e.* RER values near unity indicate carbohydrate usage, while lower values (0.9−0.7) indicate lipid use. For CLAMS analysis eight mice per group were single-housed for 48 hours in the system. In order to minimize the stress of single housing the animals for the duration of the metabolic assessment, testing animals were housed individually for 24 hours prior to the start of the test.

### Measurement of glucose, lipids and hormone levels

Both fasting and non-fasting glucose levels were measured using the EasyGluco blood glucose monitoring system as described previously (US Diagnostics, Inc., New York, NY) [37], [39]. For fasting glucose measurements, mice were food deprived 12 hours prior to blood collection. Plasma insulin, leptin, GIP (total), PP and PYY were measured using the Linco-Millipore multiplex kit according to the manufacturer's instructions (Millipore, Billerica MA). Briefly, plasma samples were incubated together with antibody-conjugated beads for 16 hours at 4°C. After three washes, a biotinylated detection antibody was added. Samples were then incubated for 1 hour at room temperature and streptavidin-horseradish peroxidase added. Fluorescent signals were detected using a Bio-Plex 200 suspension array system (Bio-Rad, Hercules, CA). Hormone levels were derived by interpolation from a reference curve generated in the same assay with reference standards of known concentrations of the detected hormones. Plasma HDL, LDL, total cholesterol and triglycerides were measured enzymatically using Wako Diagnostics assay kits as described previously (Wako Diagnostic, Richmond, VA) [40]. Final experimental concentrations were derived by interpolation from a reference curve generated in the same assay with reference standards of known concentrations of the detected lipids. At least 8 animals were included in each group.

### Immunohistochemistry

Immunofluorescence (IF) analyses were performed using antigen retrieval (1x citrate buffer (Biogenex, San Ramon, CA) at 98°C for 20 minutes) as described previously [31], [41], [42]. Cryostat sections were blocked in 5% bovine serum albumin (BSA; Sigma) and 0.1% Tween-20 in 1x Tris-buffered saline (TBS) (pH 7.4) for one hour at room temperature, followed by incubation in a specific primary antibody in 1% BSA and 0.1% Tween-20 in TBS (pH 7.4) overnight at 4°C. Sources and dilutions of the applied primary antibodies are listed in Table 1. After washing, sections were incubated for 1 hour in fluorescent secondary antibodies (1∶1000 dilutions; Invitrogen, Grand Island, NY) along with DAPI (1∶5000 dilution; Invitrogen) for nuclear staining. No fluorescent staining was observed in any sections when the primary antibodies were omitted.

**Table 1:** Primary antibodies used in immunofluorescence analyses.

Antigen	Host	Vendor	Dilution
Nucleoside triphosphate diphosphohydrolase-2 (NTPDase2)	Rabbit	http://www.ectonucleotidases-ab.com	1∶1000
Phospholipase C_β_2 (PLCβ2)	Rabbit	Santa Cruz Biothechnology Inc	1∶200
Neural Cell Adhesion Molecule (NCAM)	Rabbit	Millipore	1∶500
Sonic Hedgehog (Shh)	Rabbit	Santa Cruz Biothechnology Inc	1∶100
Glucagon-like peptide-1 (GLP-1)	Mouse	USBiological	1∶100
Leptin Receptor (Ob-Rb)	Goat	Abcam	1∶500
Anti-Epithelial Sodium Channel-gamma (ENaCγ)	Rabbit	Millipore	1∶100
GPR120	Rabbit	MBL International Corporation	1∶200
CD36	Mouse	BD Biosciences	1∶100
Ghrelin	Rabbit	Phoenix Pharmaceuticals	1∶200

Antigen, host, vendor, and dilution used are listed for all primary antibodies used in immunoflorescence analyses.

### Quantification of immunoreactive taste cells

In order to obtain consistent tongue section samples, without bias throughout the papillae, each papilla was systematically sectioned and every tenth section was saved onto a slide. As taste buds are approximately 80–100 µm in length, sampling every tenth section ensured that no two sections were likely to be collected from the same taste bud. Confocal images were collected using an LSM-710 confocal microscope (Carl Zeiss MicroImaging, Thornwood, NY) in single planes. Approximately 100–120 taste buds per group were analyzed as described previously [31], [33], [35]. Cells were scored as immunopositive only if a nuclear profile was present within the cell. The total number of cells in the section was determined by counting the number of DAPI stained nuclei present in each taste bud. Finally, the percentage of immunopositive taste cells was calculated by dividing the number of immunopositive taste cells by the total number of the taste cells in each taste bud. Both image capture and data analysis were performed by trained researchers who were blind to the experimental and control conditions.

### Real-time RT-PCR

Real-time RT-PCR experiments were performed on total RNA isolated from taste buds of circumvallate papillae as described previously [43], [44]. Briefly, approximately 1 ml of enzyme solution consisting of 2.0 mg/ml elastase and 2.0 mg/ml dispase dissolved in physiological saline (120 mM NaCl, 20 mM KCl, 10 mM HEPES, 2 mM BAPTA, pH 7.4) was injected between the muscle layers and the epithelium of the tongue. After 30 min incubation, the entire posterior tissue was peeled away under a dissecting microscope. Lingual epithelium was gently agitated in fresh saline and dissociated taste buds were harvested and collected into microtubes containing TRIzol reagent (Invitrogen Life Technologies). After isolation, RNA was treated with DNase I (Invitrogen Life Technologies) and then was used to synthesize the first strand cDNA. A two step real-time reverse transcription (RT) was performed to reverse transcribe total RNA into cDNA as described previously [31], [34]. Next, PCR was carried out using gene-specific primer pairs (Table 2) and SYBR Green PCR master mix (Applied Biosystems) in ABI prism 7300 sequence detection system (Applied Biosystems). The amplification conditions were 50°C (2 min), 95°C (10 min), and then 40 cycles at 95°C (15 s) and 60°C (1 min). The data were normalized to glyceraldehyde 3-phosphate dehydrogenase (Gapdh) mRNA. All real-time PCR analyses are represented as the mean ± S.E.M. from at least three independent experiments, each performed in triplicate.

**Table 2:** Primers for real-time RT-PCR.

Gene name	Forward Primers (5′--3′)	Reverse Primers (5′--3′)
Cd36	GATGACGTGGCAAAGAACAG	TCCTCGGGGTCCTGAGTTAT
Gpr120	ACCAAGTCAATCGCACCCAC	GTGAGACGACAAAGATGAGCC
Trpm5	CCAGCATAAGCGACAACATCT	GAGCATACAGTAGTTGGCCTG
Tas1r1	CTGCCAAAGGACAGAATCCTC	GAACCGCATGGCTTGGAAG
Tas1r3	TGGGGGCCTCTTTGTGTCT	TGGGTTGTGTTCTCTGGTTGA
Gapdh	AGGTCGGTGTGAACGGATTTG	TGTAGACCATGTAGTTGAGGTCA

Forward and reverse primers used in quantification of gene expression levels of multiple taste related genes.

### Statistical Analyses

All data represent means ± S.E.M. from at least three independent experimental replicates. Error bars represent the ±95% confidence interval. ANOVA was performed by GraphPad Prism (version 5.0) as appropriate. *p*<0.05 was considered statistically significant throughout the study.

## Results

### Ghrelin^−/−^ and GOAT^−/−^ mice possess distinct patterns of body composition and metabolic hormone levels

**Figure 1:**
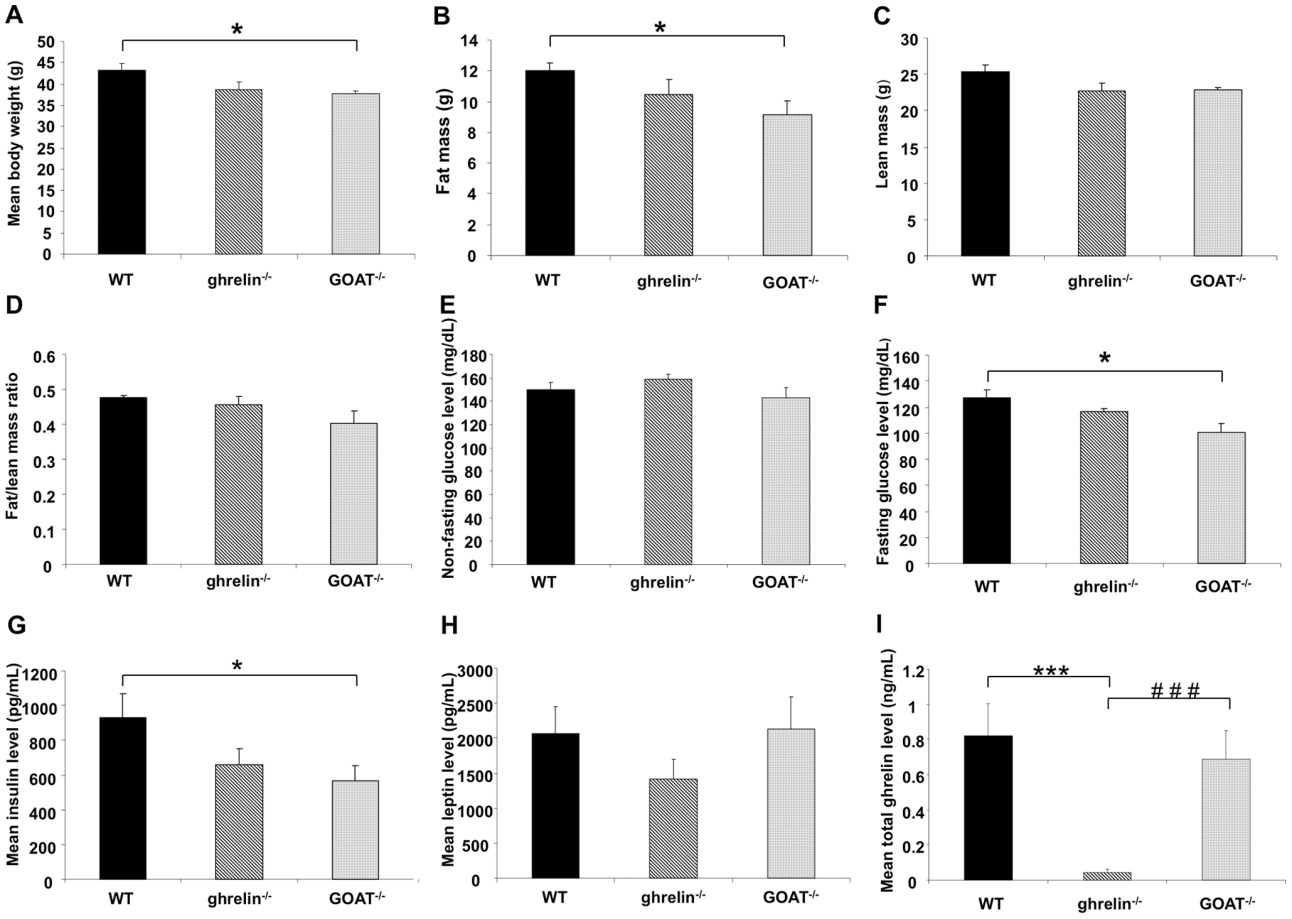
Body weight, body mass composition and metabolic hormone alterations of ghrelin^−/−^ and GOAT^−/−^ mice. (A) mean body weight, (B) fat mass, (C) lean mass, (D) fat/lean ratio of wild-type (WT), ghrelin^−/−^ and GOAT^−/−^ mice. Both non-fasting (E) and fasting (F) glucose levels were measured in the wild-type (WT), ghrelin^−/−^ and GOAT^−/−^ mice. Plasma insulin (G) and leptin (H) levels were measured using the Millipore mouse gut hormone multiplex kit. (I) Total ghrelin levels in the wild-type (WT), ghrelin^−/−^ and GOAT^−/−^ mice. Values are expressed as means ± SEM. *p≤0.05 versus WT; ***p≤0.001 versus WT; ### *p*≤0.001 versus ghrelin^−/−^, n = 8–10/group.

GOAT^−/−^, but not ghrelin^−/−^, mice demonstrated a significantly reduced bodyweight (Fig. 1A) and fat mass (Fig. 1B) compared to WT controls. No significant difference of lean mass or fat/lean mass ratio was observed between the experimental groups (Fig. 1C-D). Neither ghrelin^−/−^ nor GOAT^−/−^ mice demonstrated any difference in fasting glucose levels compared to WT mice (Fig. 1E). Compared to WT controls, GOAT^−/−^ mice possessed significantly reduced fasting glucose levels (Fig. 1F) and insulin levels (Fig. 1G), while ghrelin^−/−^ mice demonstrated a similar but non-significant trend (Fig. 1F-G). Leptin levels were not significantly different between WT and ghrelin^−/−^ or GOAT^−/−^ mice (Fig. 1H). As expected, total circulatory ghrelin was virtually undetectable in the ghrelin^−/−^ mice while present at nearly WT levels in GOAT^−/−^ mice (Fig. 1I).

### Whole-animal metabolic and behavioral activity in ghrelin^−/−^ and GOAT^−/−^ mice reveals differences in food and water ingestion

**Figure 2:**
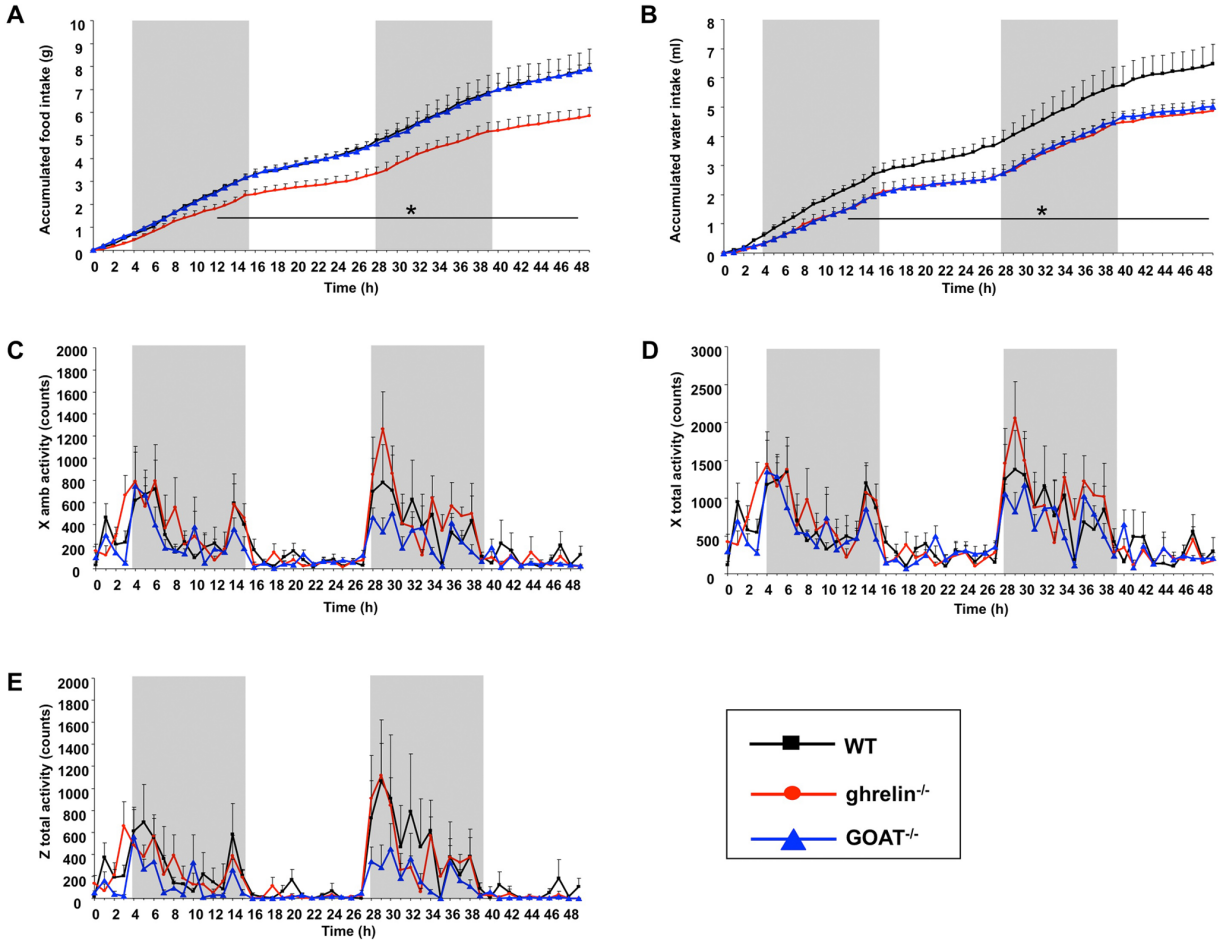
Physiological parameters of wild-type (WT), ghrelin^−/−^ and GOAT^−/−^ mice. Accumulated food (A) and water (B) intake were measured using the Comprehensive Lab Animal Monitoring System (CLAMS). There was no difference in X ambulatory (C), X total (D) and Z total activity (E) between wild-type (WT), ghrelin^−/−^ and GOAT^−/−^ mice. Grey bars indicate dark cycles. Datapoints are expressed as means ± S.E.M. *p≤0.05 versus WT. n = 6–8/group.

Comprehensive metabolic profiles of WT, ghrelin^−/−^, and GOAT^−/−^ mice were obtained using the CLAMS system. Ghrelin^−/−^, but not GOAT^−/−^, mice exhibited a significant reduction of accumulated food intake compared to WT mice (Fig. 2A). Both ghrelin^−/−^ and GOAT^−/−^ mice showed significantly decreased water intake compared with WT controls (Fig. 2B). No significant differences in activity were found including x ambulatory, x total and z total activity between all three mouse test groups (Fig. 2C-E).

**Figure 3:**
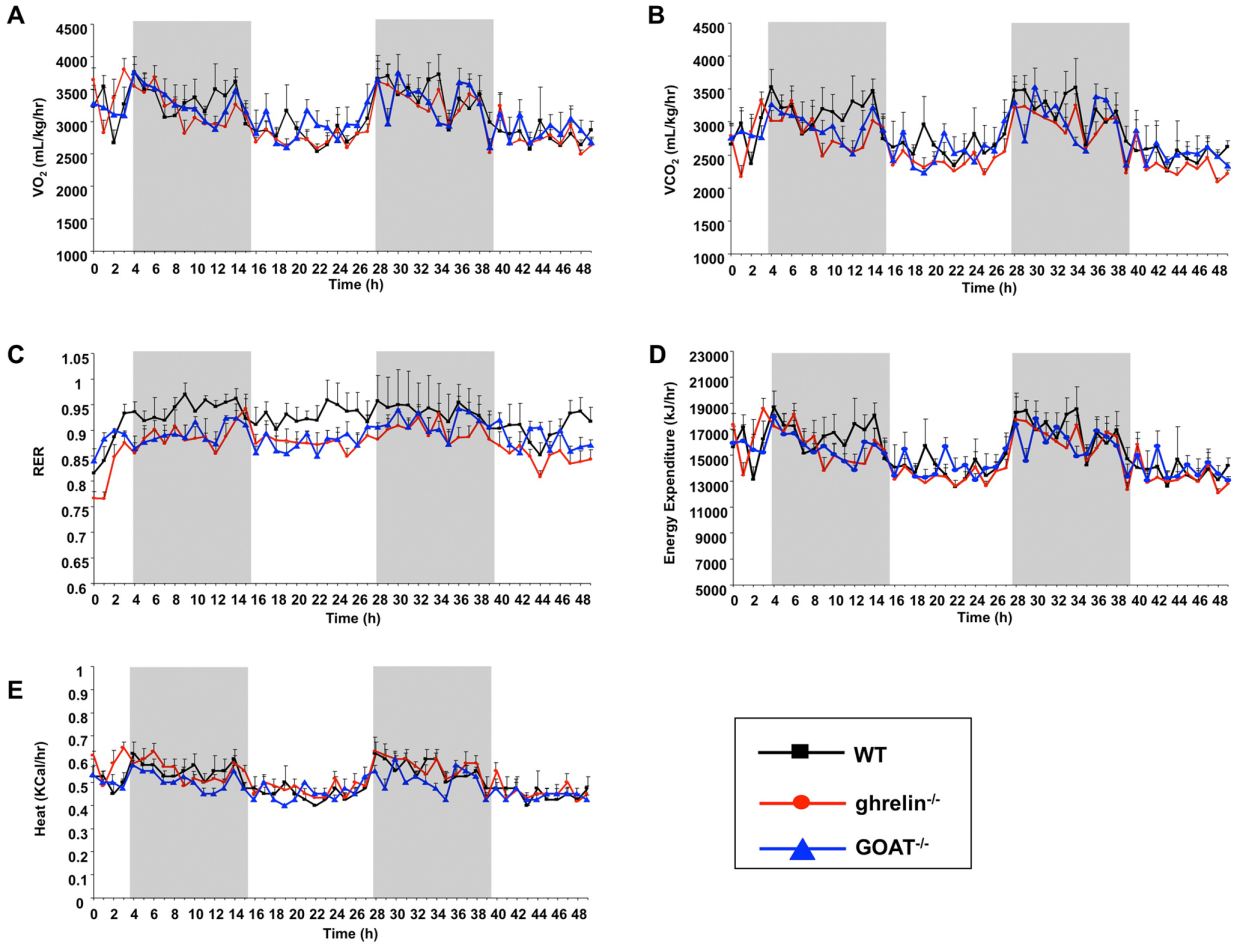
Metabolic parameters of wild-type (WT), ghrelin^−/−^ and GOAT^−/−^ mice. Oxygen consumption (A), carbon dioxide production (B), respiratory exchange ratio (C), energy expenditure (D) and heat production (E) were measured using the Comprehensive Lab Animal Monitoring System (CLAMS). Grey bars indicate dark cycles. Datapoints are expressed as means ± S.E.M. n = 6–8/group.

Ghrelin^−/−^ and GOAT^−/−^ mice displayed similar oxygen consumption (Fig. 3A) and carbon dioxide production (Fig. 3B) profiles. There was a non-significant trend for reduced respiratory exchange ratio (RER) in the ghrelin^−/−^ and GOAT^−/−^ mice compared to WT mice (Fig. 3C). Ghrelin^−/−^ and GOAT^−/−^ mice demonstrated similar levels of energy expenditure (Fig. 3D) and heat production (Fig. 3E) compared to WT control mice.

### Ghrelin^−/−^ but not GOAT^−/−^ mice exhibit elevated plasma triglycerides and ketone body levels

**Figure 4:**
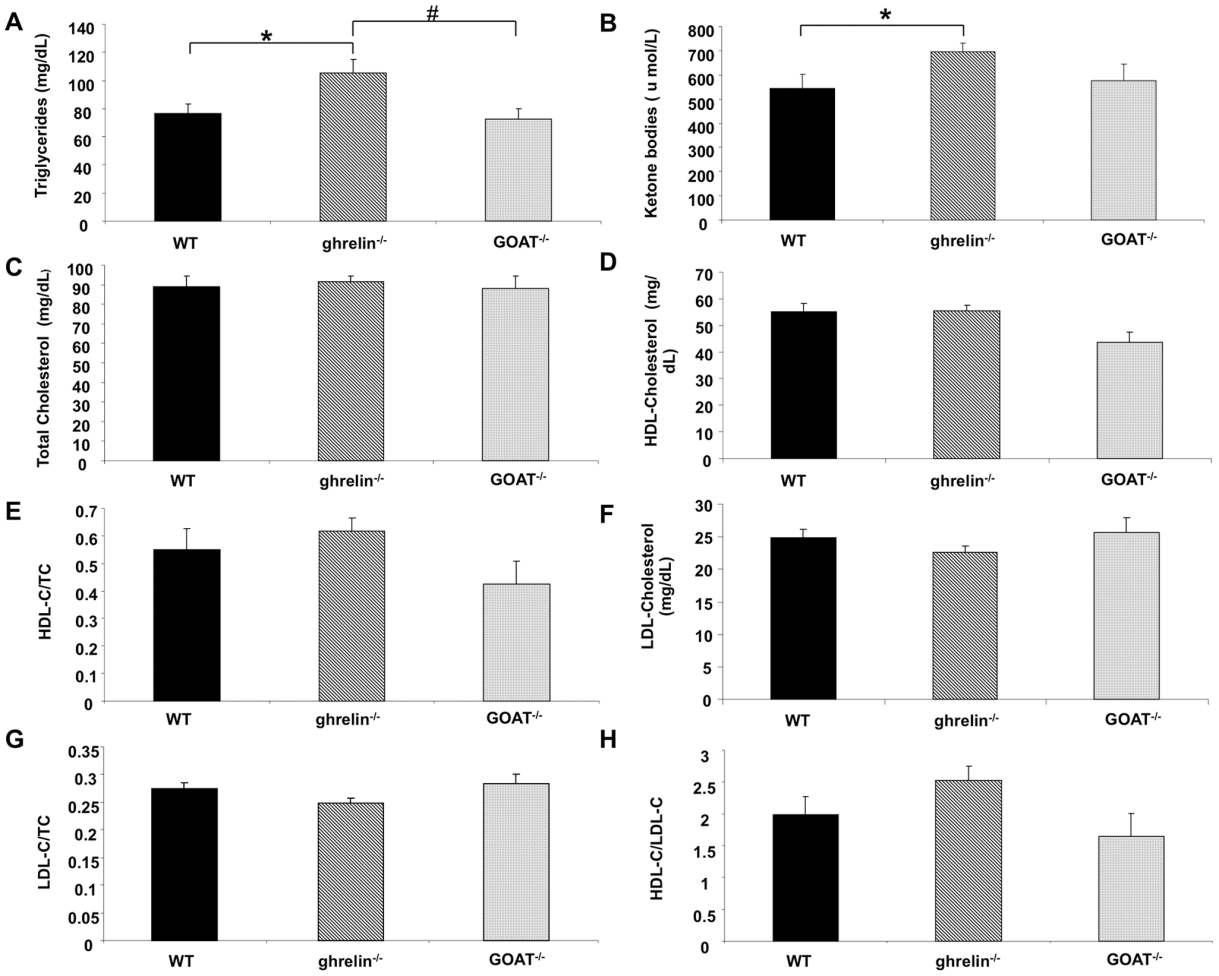
Lipid profiles of wild-type (WT), ghrelin^−/−^ and GOAT^−/−^ mice. Plasma total triglycerides (A), ketone bodies (B), cholesterol (C), HDL-C (cholesterol) (D), and LDL-C (cholesterol) (F) levels were measured using Wako Diagnostics assay kits and HDL-C/TC (total cholesterol) (E), LDL-C/TC (total cholesterol) (G), and HDL-C/LDL-C ratios (H) were calculated. Values are expressed as means ± SEM. *p≤0.05 versus WT; #*p*≤0.05 versus ghrelin^−/−^, n = 8–10/group.

Compared with WT mice, ghrelin^−/−^, but not GOAT^−/−^, mice possessed increased plasma levels of triglycerides (Fig. 4A) and ketone bodies (Fig. 4B). We observed no significant difference in the levels of total cholesterol (TC) (Fig. 4C), HDL-cholesterol (Fig. 4D), the HDL-C/TC ratio (Fig. 4E), LDL-cholesterol (Fig. 4F), the LDL-C/TC ratio (Fig. 4G) and the HDL-C/LDL-C ratio (Fig. 4H) between ghrelin^−/−^ or GOAT^−/−^ mice and WT controls.

### Altered salt and lipid taste preference in ghrelin^−/−^ and GOAT^−/−^ mice

**Figure 5:**
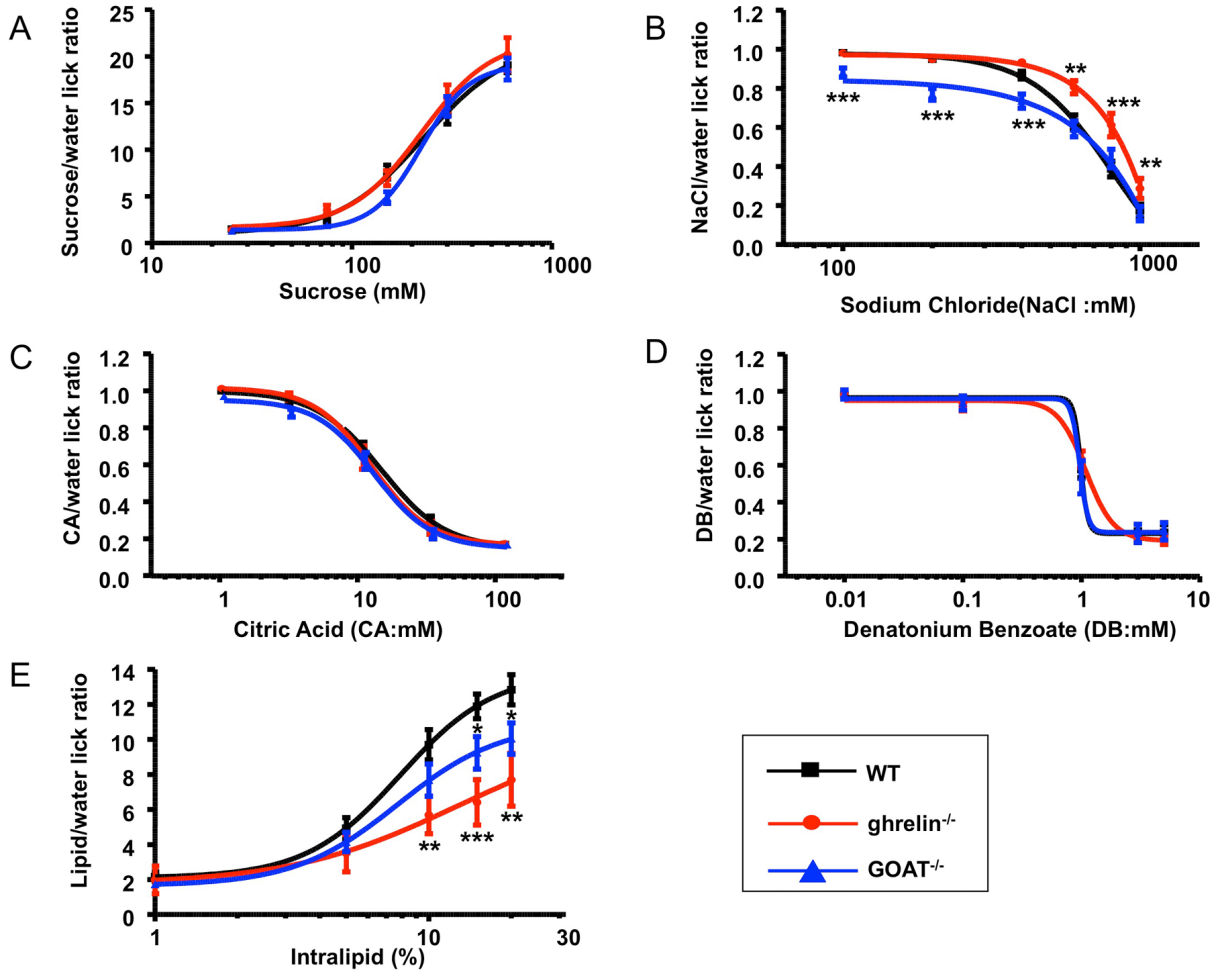
Altered salt and lipid taste sensitivity in ghrelin^−/−^ and GOAT^−/−^ compared to wild-type mice. Taste responses, expressed as tastant/water lick ratios and as a function of stimulus concentration, of wild-type (WT, black), ghrelin null (red) and GOAT null (blue) mice to sucrose (A), NaCl (B), citric acid (C), denatonium benzoate (D), and intralipid tastants (E). Datapoints are expressed as means ± S.E.M. Response curves were fit as described in the Methods section. *p≤0.05, **p≤0.01, ***p≤0.001 versus WT, n = 6–8/group.

We have previously shown that both ghrelin and GOAT are expressed in taste cells. We therefore investigated whether genomic ablation of ghrelin or GOAT would exert any specific effects upon the major taste modalities. We tested the sensitivity of ghrelin^−/−^ and GOAT^−/−^ mice to sweet (sucrose), salty (sodium chloride), sour (citric acid), bitter (denatonium benzoate) and lipid (intralipid) tastants. Using a brief-exposure gustometer test we found no changes in sweet taste sensitivity in ghrelin^−/−^ or GOAT^−/−^ mice compared to WT control mice (Fig. 5A). In contrast, the aversive salt taste perception was differentially affected in ghrelin^−/−^ (attenuated) and GOAT^−/−^ (enhanced) mice compared to WT controls (Fig. 5B). Ghrelin^−/−^ and GOAT^−/−^ mice possessed similar sour (Fig. 5C) and bitter (Fig. 5D) taste responsivities compared to WT mice. With respect to lipid tastant sensitivity, both ghrelin^−/−^ and GOAT^−/−^ mice exhibited significantly reduced sensitivity to the appetitive lipid stimuli (Fig. 5E).

### Gross taste bud morphology and taste cell composition is not significantly altered in ghrelin^−/−^ or GOAT^−/−^ mice

**Figure 6:**
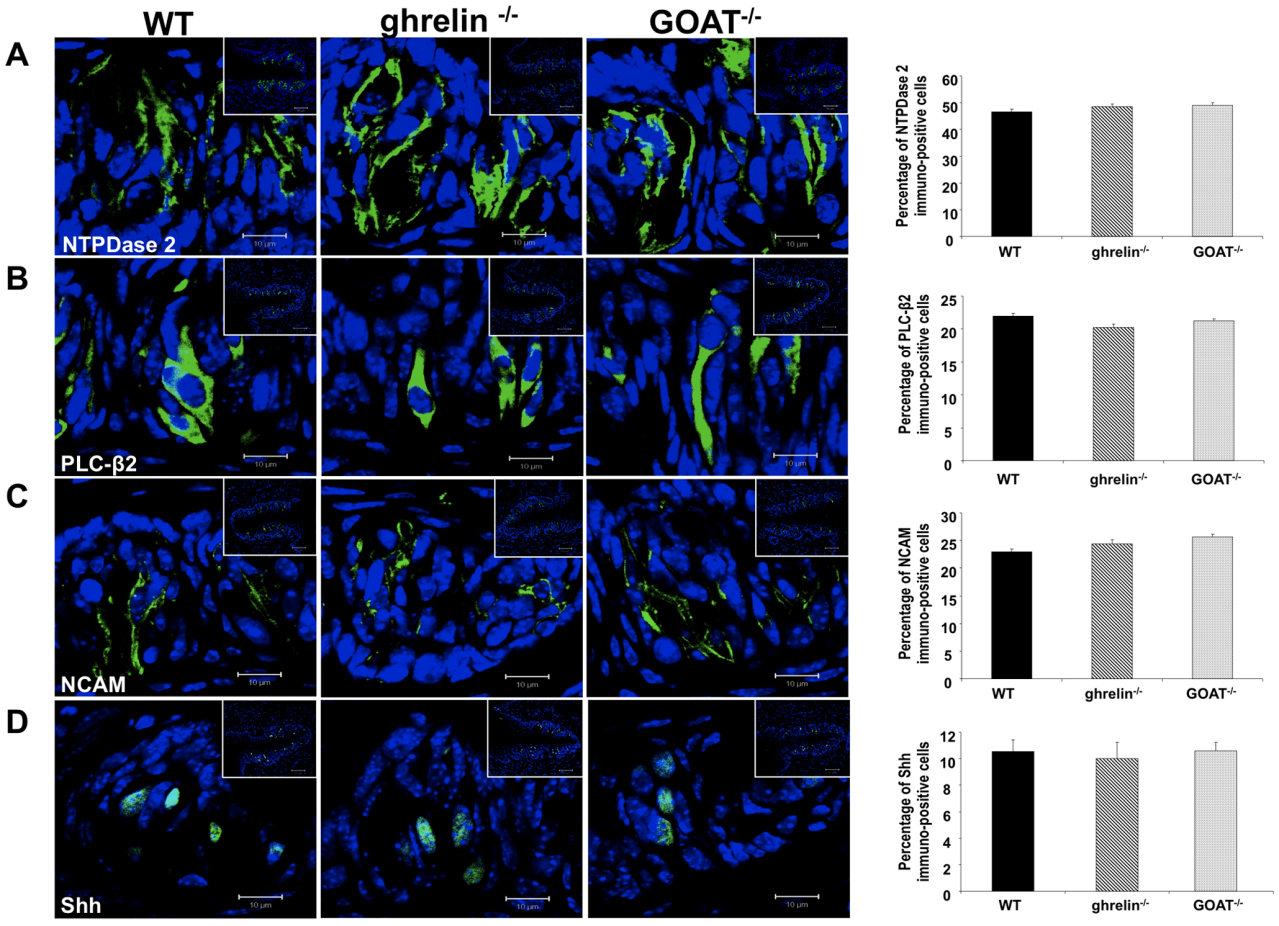
Expression of taste cell markers in circumvallate papillae of wild-type (WT), Ghrelin^−/−^ and GOAT^−/−^ mice. (A), (B), (C), and (D) are high magnification representative fluorescent images of the different taste cell markers (NTPDase2, PLCβ2, NCAM, and Shh). The inset boxes in each image indicate a low magnification field view of the circumvallate papillae. The histograms associated with each taste bud figure represent the percentage of cells containing each marker out of the total number of cells in each taste bud. Values are expressed as means ± SEM. n = 3–4/group. Bars in each high magnification image are 10 µm; bars in each inset box are 50 µm.

As both ghrelin and GOAT genomic deletions affected two perceptive taste modalities (salty and lipid) we next assessed the gross morphology of the taste buds in these mice. Ghrelin^−/−^ and GOAT^−/−^ mice demonstrated similar taste bud size and taste cell number per taste bud compared to WT control mice (Fig. S1 A, B). Upon investigation of taste cell type composition of the taste buds in ghrelin^−/−^ and GOAT^−/−^ mice we found, compared to WT mice, no significant changes in the numbers of Type I (NTPDase2 positive: Fig. 6A), Type II (PLC-β2 positive: Fig. 6B), Type III (NCAM positive: Fig. 6C) or Type IV (Shh positive: Fig. 6D) taste cells.

### Ghrelin^−/−^ and GOAT^−/−^ mice demonstrate reduced expression levels of lipid- and salt taste-modulating factors

**Figure 7:**
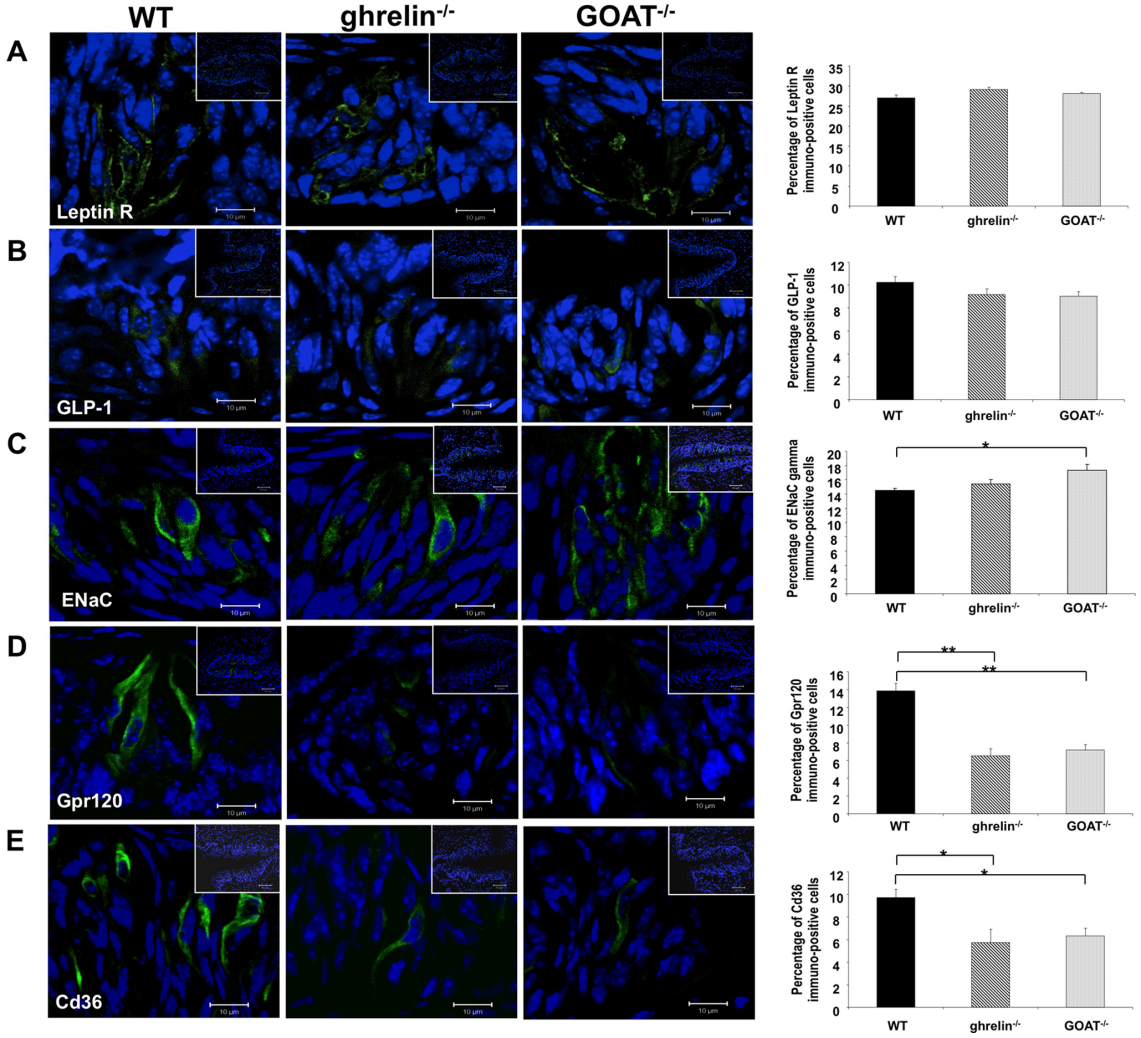
Expression of taste-modulating factors in circumvallate papillae of wild-type (WT), ghrelin^−/−^ and GOAT^−/−^ mice. (A), (B), (C), (D), and (E) are representative high magnification immunofluorescent images of leptin receptor (R), GLP-1, ENaC□, Gpr120, and Cd36, respectively. The inset boxes in each image indicate a low magnification field view of the circumvallate papillae. The histograms associated with each taste bud figure represent the percentage of the immunopositive cells out of the total number of cells in each taste bud. Blue  =  DAPI nuclear stain. Values are expressed as means ± SEM. *p≤0.05, **p≤0.01 versus WT, n = 3–4/group. Bars in each high magnification image are 10 µm; bars in each inset box are 50 µm.

As we found no significant alteration in total taste cell type number we next investigated the expression of multiple taste-modulatory factors in the taste cells of ghrelin^−/−^ and GOAT^−/−^ mice. As expected from our previous gustometer data (Fig. 5A), we found no significant differences in the expression of leptin receptor and glucagon-like peptide-1 (GLP-1), two sweet-taste related factors, between WT and ghrelin^−/−^ or GOAT^−/−^ mice (Fig. 7A, B). In the taste buds of circumvallate papillae, GOAT^−/−^ mice demonstrated a significantly higher expression of the salt taste-modulating factor, ENaC□, compared to WT mice (Fig. 7C). Ghrelin^−/−^ mice however exhibited comparable expression levels of ENaC□ with WT mice (Fig. 7C). A similar alteration pattern was also observed in the taste buds of fungiform and foliate papillae, in which ENaC□ expressing cells were increased in GOAT^−/−^ while comparable expression levels were observed in ghrelin^−/−^ compared to WT mice (Fig. S3). In contrast both ghrelin^−/−^ and GOAT^−/−^ mice displayed significantly decreased taste cell expression of Gpr120 and Cd36, two lipid taste-related factors, compared to WT mice (Fig. 7D, E). The mRNA expression levels of Cd36, Gpr120, Trpm5, T1rR1 and T1rR3 were also examined in wild-type, ghrelin^−/−^ and GOAT^−/−^ mice taste buds. Compared to the wild-type mice, the mRNA levels of Cd36 and Gpr120 were significantly reduced in both ghrelin^−/−^ and GOAT^−/−^ mice. However the mRNA expression of Trpm5, T1r1 and T1rR3 was not significantly altered in ghrelin^−/−^ and GOAT^−/−^ mice compared to wild-type mice (Figure S4).

## Discussion

In the present study we attempted to assess whether the presence of a functional ghrelin/GOAT system in the tongue plays a modulatory role in taste sensitivity. Recent experimental data has begun to demonstrate that taste perception capacity is tightly linked to metabolic status [33], [45], [46]. Therefore in ghrelin^−/−^ and GOAT^−/−^ mice we first assessed their gross metabolic status compared to age-matched WT controls (Table 3). No significant differences in body weight, fat composition and glucose control were observed in ghrelin^−/−^ mice compared to WT controls (Fig. 1). Our findings mirror those of other research groups in that, on a normal chow diet, ghrelin^−/−^ mice exhibit normal size, body composition, food intake, energy expenditure, serum chemistry, glucose, insulin, and leptin levels [47], [48]. Therefore it appears that there may be a significant degree of developmental compensation in the case of genomic ghrelin ablation. In some cases however, ghrelin^−/−^ mice have demonstrated reduced body weight, fat mass, and improved serum chemistry profile on high-fat diets [49], [50], [51]. In addition, a recently published paper demonstrated that the ablation of ghrelin receptor (growth hormone secretagogue receptor, GHS-R) reduced glucose/lipid intake and body fat which is different from the phenotype of ghrelin null mice [52] while another group reported that ghrelin receptor (GHS-R) null mice showed reduced fat/lean ratio and increased energy expenditure compared to wild-type mice yet no significant differences were observed in ghrelin knockout (Ghrl^−/−^) mice. The results of this study could indicate that there are other ghrelin subtype receptor(s) apart from GHS-R and other as-yet-unidentified GHS-R ligand(s) besides ghrelin capable of inducing changes in metabolic functionality [53]. Although O-n-octanoylation by GOAT at serine 3 is important for acyl ghrelin binding to its receptor GHS-R1a, the mouse lines with genetic deletion of ghrelin and GOAT exhibited differential phenotypes [54]. There are several potential reasons to explain why ghrelin and GOAT null mice are capable of exhibiting these differential phenotypes. Firstly, though GOAT does display consistent overlap in its expression pattern with that of ghrelin, GOAT is uniquely expressed in some tissues which could suggest that additional peptide(s) may be acylated by GOAT in addition to ghrelin [54]. Thus the genetic deletion of GOAT may have some effects on other as-yet-unidentified GOAT substrates, and these as-yet-unidentified alterations could be affecting the differential metabolic phenotypes we and others observed. Secondly, accumulating evidence suggests that des-acyl ghrelin (DAG), which represents approximately 90% of total ghrelin detected in the circulation, might be a functional inhibitor of ghrelin and it also seems to have acyl ghrelin (AG)-independent effects [27], [55]. The evidence that GOAT gene disruption in mouse models completely abolishes ghrelin acylation without affecting circulating des-acyl ghrelin levels suggests that GOAT null mice may exhibit, at least to some extent, different phenotypes from ghrelin null mice which have none of the various forms of ghrelin [18]. In addition, the third ghrelin gene product, obestatin, was reported to play a role in adipocyte function and glucose metabolism, though its effect on food intake remains debatable [56]. However, the effect of obestatin on adipocyte function and glucose metabolism in GOAT knockout mice is worth further investigation.

**Table 3:** Summary of phenotypes of ghrelin^−/−^ and GOAT^−/−^ mice compared to wild-type (WT) mice.

	ghrelin^−/−^ mice (vs. WT mice)	GOAT^−/−^ mice (vs. WT mice)
Mean body weight	Comparable	Decreased
Fat mass	Comparable	Decreased
Lean mass	Comparable	Comparable
Fasting glucose level	Comparable	Decreased
Fasting insulin level	Comparable	Decreased
Leptin level	Comparable	Comparable
Total ghrelin level	None	Comparable
Food intake	Decreased	Comparable
Water intake	Decreased	Decreased
Activity	Comparable	Comparable
Energy expenditure	Comparable	Comparable
Triglycerides	Increased	Comparable
Ketone bodies	Increased	Comparable
Cholesterol	Comparable	Comparable
Sweet (sucrose) taste responsivity	Comparable	Comparable
Salty (sodium chloride) taste responsivity	Attenuated	Enhanced
Sour (citric acid) taste responsivity	Comparable	Comparable
Bitter (denatonium benzoate) taste responsivity	Comparable	Comparable
Lipid (intralipid) taste responsivity	Attenuated	Attenuated

Phenotypic summary includes all metabolic data as well as taste responsivity data as obtained by comparing ghrelin ^−/−^ mice and GOAT ^−/−^ mice each to WT mice.

In our study ghrelin^−/−^ mice exhibited reduced food intake (Fig. 2A) yet also simultaneously exhibited increased plasma triglycerides levels (Fig. 4A). The release of free fatty acids from plasma triglycerides (TGs) can provide postprandial satiety signals to the central nervous system [57]. Therefore the higher triglyceride levels in the ghrelin^−/−^ mice may elicit satiety signals, contributing as a negative feedback to the decreased food intake observed in ghrelin^−/−^ mice. GOAT^−/−^ mice did not demonstrate these changes in food intake or TG levels, therefore indicating a potential divergence in AG and DAG functionality in this specific satiety system. In subsequent two-bottle taste test experiments GOAT^−/−^ mice showed a greater preference to the lipid solution compared to ghrelin^−/−^ mice (Fig. S2). This data indeed suggests a potential satiety difference in these two models as for two-bottle taste testing the preference data is generated by the combinational result of post-ingestive effects of lipid on whole body metabolism and satiety as well as initial tastant perceptive ability. In addition, both ghrelin^−/−^ and GOAT^−/−^ mice demonstrated decreased water intake (Fig. 2B). In accordance with this there is evidence showing that ghrelin is also involved in the regulation of water balance using exogenously administrated ghrelin animal models [58], [59].

Our study is the first report that demonstrates that ghrelin^−/−^ and GOAT^−/−^ mice exhibit significantly decreased taste sensitivity to lipid stimuli, thereby implicating the ghrelin/GOAT system in the regulation of lipid taste sensation. Ghrelin acylation, which is dependent on the function of GOAT, and the availability of substrates such as proghrelin and short- to medium-chain fatty acids is required for the binding and activation of the classical ghrelin receptor, GHS-R1a. GOAT gene disruption in mouse models completely abolishes ghrelin acylation, without affecting circulating des-acyl ghrelin levels [18]. Consistent with this, we have demonstrated that none of the various forms of ghrelin are detectable in the ghrelin^−/−^ mice, but GOAT^−/−^ mice possess comparable total ghrelin levels with WT mice (Fig. 1I, S1 C-F). The ghrelin found in GOAT^−/−^ mice is most likely constituted of des-acyl ghrelin, unacylated ghrelin and other forms of ghrelin. As both ghrelin^−/−^ and GOAT^−/−^ mice possessed reduced lipid taste sensitivity, this suggests that acylated ghrelin may be strongly involved in lipid taste perception.

Compelling evidence suggests that fat taste sensation is an important taste modality in mammals [11], [60], [61]. Spontaneous preference for fatty food is a common trait in both animals and humans [62], [63], however overconsumption of fatty food can easily lead to obesity and obesity-related diseases [64]. Multiple factors account for the spontaneous fat preference, such as taste of fatty food, texture of lipid, somesthesic and olfactory cues from lipid, as well as feedback from internal homeostatic regulatory functions. We observed that ghrelin^−/−^ and GOAT^−/−^ mice possessed reduced responsivity to the fat emulsion made up of predominantly unsaturated long-chain (number of carbons ≥16) fatty acids (LCFA), which is highly preferred by normal rodents. An early study contrasted responses of male rats to distinct fatty acids and found that rats preferred LCFA fluids but tended to avoid caprylic acid, the medium-chain fatty acid [12]. An oral lipid load was found to be sufficient to enhance the protein content of pancreatobiliary juice in esophagostomized rats [65]. In rats with an esophageal ligation to prevent nutrient ingestion, oral exposure with linoleic, linolenic, and oleic fatty acids was found to augment pancreatic exocrine secretion whereas no change occurred following oral exposure to the medium-chain fatty acid, caprylic acid (C8:0), indicating that medium-chain fatty acids may not be detected orally [65], [66].

It has been demonstrated that the glycoprotein Cd36, mainly expressed in circumvallate papillae of the tongue in various species, displays a high affinity for long-chain fatty acids (LCFAs) and plays an important role in dietary fat taste perception [67]. Inactivation of the Cd36 gene has been shown to disturb the detection and consumption of LCFA in both long-term and short-term taste behavioral tests while not affecting sweet preference or bitter aversion [66], [68]. Further studies have demonstrated that the sensation of LCFA involves LCFA-induced calcium responses and the subsequent activation of gustatory neurons in a Cd36-dependent manner [67]. We found in our current study that Cd36-positive cell numbers in taste buds of ghrelin^−/−^ and GOAT^−/−^ mice were significantly reduced compared to WT mice (Fig. 7D). It is interesting to note that the expression of Cd36 has been reported to be significantly decreased in the adipose tissue of ghrelin receptor null mice [52]. The decreased Cd36-positive taste cell numbers in ghrelin^−/−^ and GOAT^−/−^ mice strongly reinforces our hypothesis that the ghrelin/GOAT system is involved in lipid sensation. In addition to Cd36, sensory G-protein-coupled receptors (GPCRs) have also been demonstrated to be involved in fatty acid-induced taste cell activation [69]. Gpr120 has been found in taste buds in various species and is mainly expressed in Type II taste cells [70], [71]. Gpr120 and a related receptor, Gpr40, have been reported to play significant roles in perception of lipids in the oral cavity. Hence Gpr40- or Gpr120-null mice possess a diminished preference for, and decreased taste nerve response to, several fatty acids [72]. However, the role of Gpr40 as a gustatory lipid sensor remains questionable because of conflicting data concerning its physical presence in taste buds [71], [73]. Our observed reduction in the number of Gpr120-positive cells in the taste buds of ghrelin^−/−^ and GOAT^−/−^ mice may contribute, to some extent, to the decreased lipid taste responsivity in ghrelin and GOAT null mice. However further work is needed to investigate other factors that affect lipid taste responsivity/sensation in the future, such as lipid-signaling transduction, gustatory nerve responses in response to lipid sensation, and the role of the central nervous system's reward system in response to lipid sensation. Ghrelin acts as a powerful regulator of serotonin levels in regions of the nucleus accumbens [74] and in addition, LCFA have been reported to induce calcium signaling and the release of neurotransmitters such as serotonin, and noradrenalin in mouse Cd36-positive taste cells [75]. Therefore it is possible that, as in the central nervous system, ghrelin may also modulate gustatory neurotransmitters involved in gustatory lipid perception. Clearly further work is needed to support such hypotheses and we will address this in further manuscripts.

It is known that there are at least two different systems involved in salt taste transduction, *i.e*. two sensory systems which are distinguished by their sensitivity or resistance to the epithelial Na^+^ channel (ENaC) blocker, amiloride [76], [77], [78]. The ENaC channel is made up of three subunits (α, β and γ); each unit is required for ENaC function [79], [80]. A recent study has demonstrated that mice lacking the ENaCα subunit in taste cells exhibit a complete loss of salt attraction and sodium taste responses [8]. We have reported previously that ghrelin is co-expressed with ENaC subunits and that GHSR^−/−^ mice possess a significantly reduced sensitivity to NaCl compared to wild-type mice [31]. In the current study, we found that ghrelin^−/−^ mice displayed decreased, while GOAT^−/−^ exhibited increased, salty taste sensitivity compared to WT mice (Fig. 5B). The significant increase in ENaC□ positive cells in the taste buds observed in this study are likely to be involved in the altered salty taste perception in GOAT^−/−^ mice. The similar behavioral reaction of salty taste sensitivity in ghrelin^−/−^ and GHSR^−/−^ mice suggests that ghrelin receptor signaling may be involved in salt taste sensitivity. However, in GOAT^−/−^ mice there may be other extant forms of ghrelin, with the exception of acylated ghrelin, that can still exert some modulatory roles in salty taste sensitivity, in either a ghrelin receptor-dependent or -independent manner. It is of course prudent to note that ghrelin^−/−^, GOAT^−/−^ and GHSR^−/−^ mice are three quite distinct murine models that may possess diverse developmental compensatory mechanisms from other hormones, family members or unknown ligands.

Ghrelin^−/−^ and GOAT^−/−^ mice both exhibited reduced lipid taste sensitivity. The most significant reduction in sensitivity was seen in ghrelin^−/−^ mice, suggesting that lipid taste sensation could be mediated naturally by multiple forms of ghrelin. In addition to this we found that ghrelin^−/−^ mice displayed decreased while GOAT^−/−^ mice exhibited increased salty taste sensitivity compared to WT control mice. The altered expression of Cd36, Gpr120 and the ENaC□ subunit in the taste buds of ghrelin^−/−^ and GOAT^−/−^ mice may be involved in the observed alterations in lipid and salt taste sensitivity, respectively. Given the evidence of a positive correlation between overall fat preference and percent body fat in human studies [81], coupled with the finding that obese patients possess a greater preference for fatty food than lean ones [63], the study of the connections between gustatory perception of dietary lipids and the regulation of eating behavior is becoming increasingly important. The presence of prepro-ghrelin, ghrelin, GHSR, and GOAT in taste cells strongly suggests that the ghrelin/GOAT system plays a local modulatory role in determining taste cell function and signaling. The altered lipid taste sensitivity in ghrelin and GOAT null mice observed in this study therefore further reinforces the posit that the ghrelin/GOAT system plays an important role in the gustatory perception of lipid as well as salty tastants. Therefore these GPCR-based signaling systems could represent important future targets for pharmacotherapeutics aimed towards the amelioration of dietary foodstuff-induced pathophysiologies such as excessive salt or fat ingestion.

## Supporting Information

Figure S1 **Analysis of gross taste bud morphology in wild-type (WT), ghrelin^−/−^ and GOAT^−/−^ mice.** (A) To calculate taste bud size, the perimeter of the taste bud (from every tenth tongue section) was outlined and the corresponding area was computed using a Zeiss LSM Image Browser. (B) The total number of cells in the section was determined by counting the number of DAPI stained nuclei present in each taste bud. (C) Immunostaining of ghrelin in the stomach of wild-type (WT) mice was employed as a positive control for the primary antibody used. Subsequent immunostaining of ghrelin in the taste buds of wild-type (WT) (D), ghrelin^−/−^ (E) and GOAT^−/−^ (F) mice, is shown respectively.
(TIF)

Figure S2 **Two-bottle taste testing for the lipid taste modality in ghrelin^−/−^ and GOAT^−/−^ mice.** 48-hour two bottle preference test of intralipid (15%) in wild-type (WT), ghrelin^−/−^ and GOAT^−/−^ mice. Bars depict the relative intralipid/water lick ratio of ghrelin^−/−^ GOAT^−/−^ mice normalized to that of wild-type mice. Values are expressed as means ± SEM. *p≤0.05 versus WT, n = 3–4/group.
(TIF)

Figure S3 **Expression of ENaC□in fungiform and foliate papillae of wild-type (WT), ghrelin^−/−^ and GOAT^−/−^ mice.** (A), ENaC□ staining (green signals) in taste buds of fungiform papillae from wild-type (WT), ghrelin^−/−^ and GOAT^−/−^ mice. (B), ENaC□ staining (green signals) in taste buds of foliate papillae from wild-type (WT), ghrelin^−/−^ and GOAT^−/−^ mice. Blue  =  DAPI nuclear stain. The histograms associated with each taste bud figure represent the percentage of the immunopositive cells out of the total number of cells in each taste bud. Values are expressed as means ± SEM. *p≤0.05 versus WT, n = 4/group. Bars in each image are 10 µm.
(TIF)

Figure S4 **Real-time RT-PCR analysis.** Relative mRNA expression of Gpr120 (A), Cd36 (B), Trpm5 (C), T1r1 (D) and T1r3 (E) was assessed by real-time RT-PCR in taste buds of wild-type (WT), ghrelin^−/−^ and GOAT^−/−^ mice. Values are expressed as means ± SEM. *p≤0.05 versus WT, n = 5/group.
(TIF)
