# Supplementary figures and images for: Altered Lipid and Salt Taste Responsivity in Ghrelin and GOAT Null Mice
Source: PLoS One. 2013 Oct 4;8(10):e76553. doi: 10.1371/journal.pone.0076553 (PMC3790684; doi:10.1371/journal.pone.0076553)

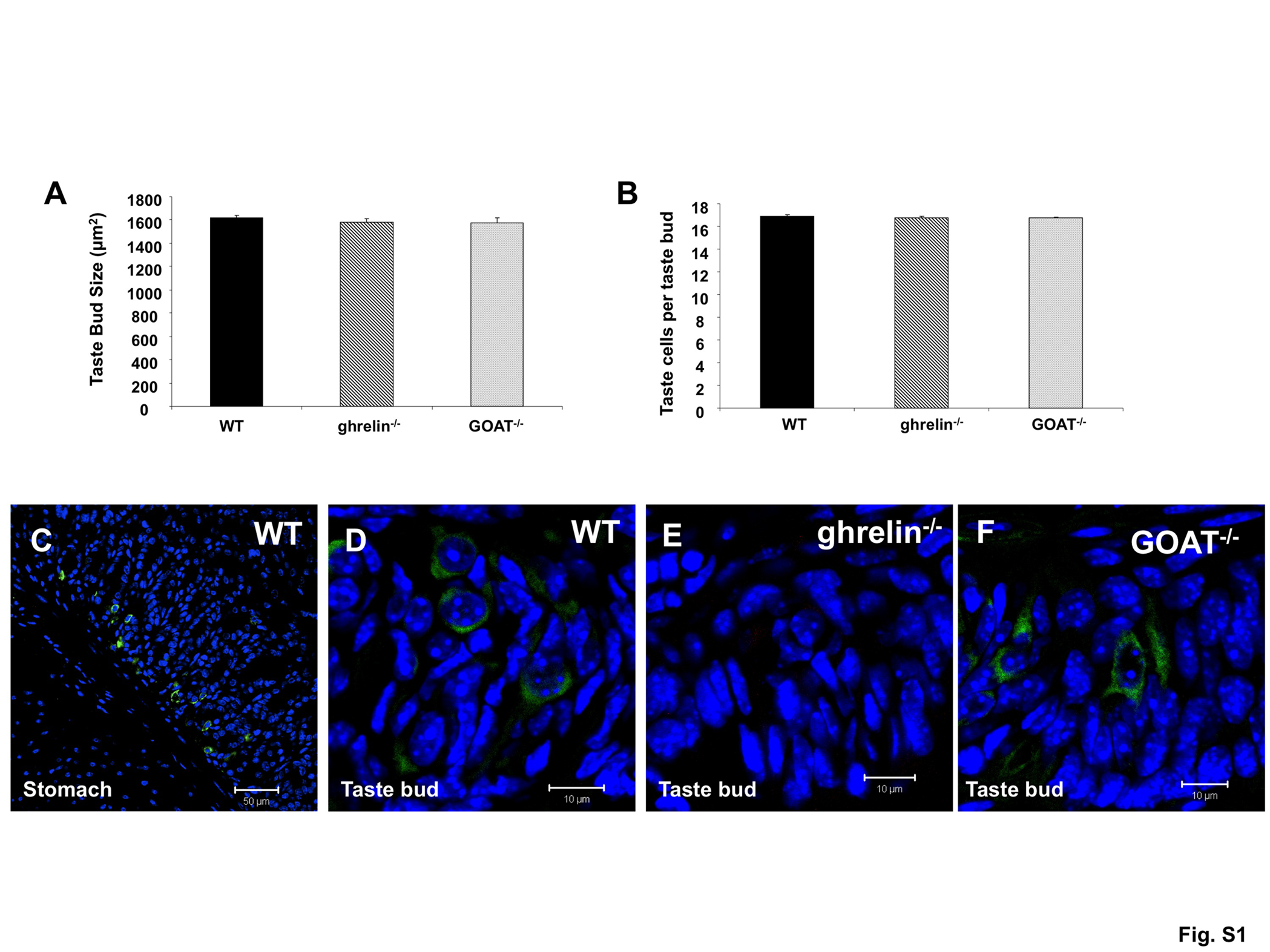

Supplement: Figure S1 — Analysis of gross taste bud morphology in wild-type (WT), ghrelin−/− and GOAT−/− mice. (A) To calculate taste bud size, the perimeter of the taste bud (from every tenth tongue section) was outlined and the corresponding area was computed using a Zeiss LSM Image Browser. (B) The total number of cells in the section was determined by counting the number of DAPI stained nuclei present in each taste bud. (C) Immunostaining of ghrelin in the stomach of wild-type (WT) mice was employed as a positive control for the primary antibody used. Subsequent immunostaining of ghrelin in the taste buds of wild-type (WT) (D), ghrelin−/− (E) and GOAT−/− (F) mice, is shown respectively. (TIF) [file pone.0076553.s001.tif]

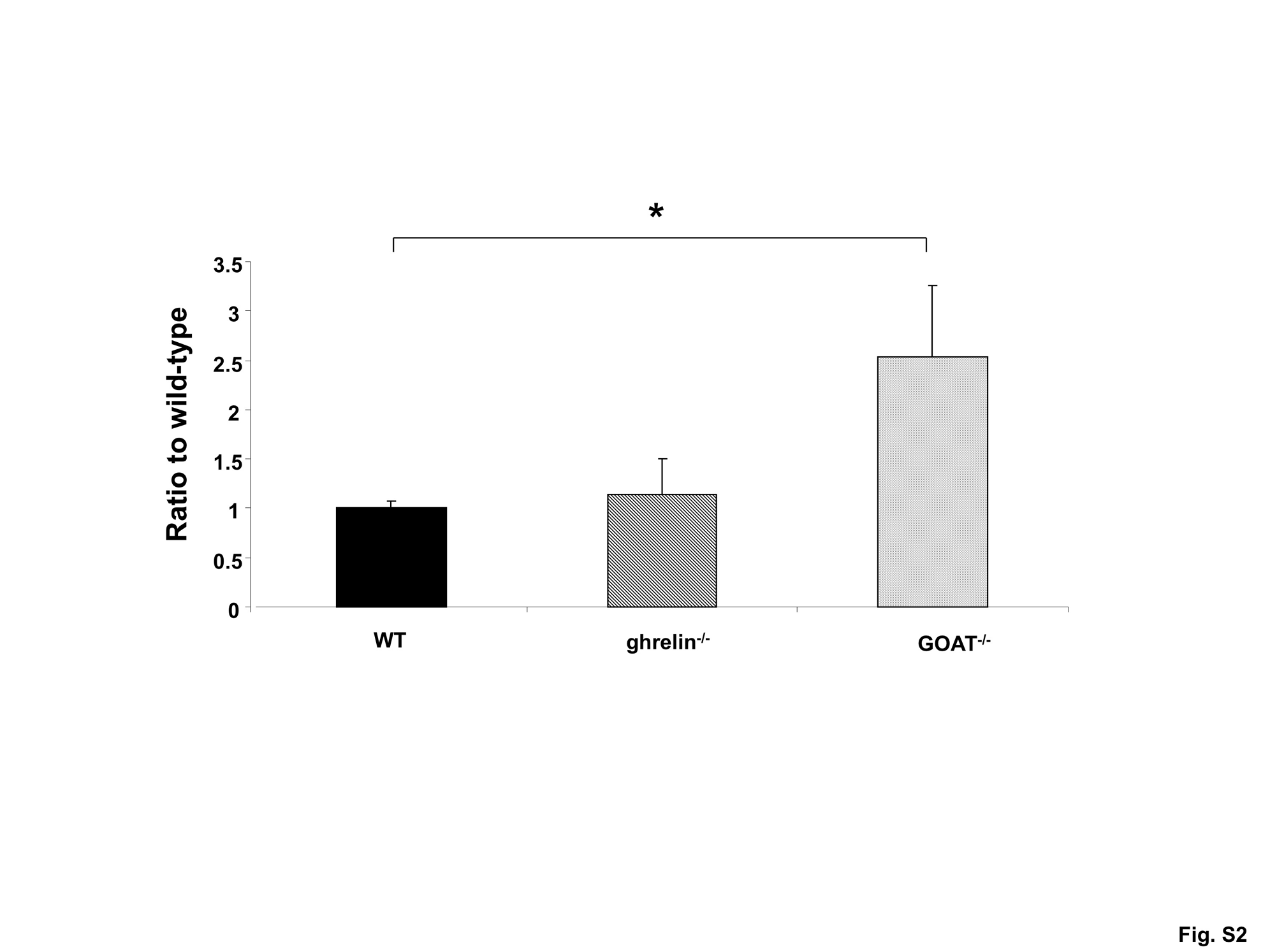

Supplement: Figure S2 — Two-bottle taste testing for the lipid taste modality in ghrelin−/− and GOAT−/− mice. 48-hour two bottle preference test of intralipid (15%) in wild-type (WT), ghrelin−/− and GOAT−/− mice. Bars depict the relative intralipid/water lick ratio of ghrelin−/− GOAT−/− mice normalized to that of wild-type mice. Values are expressed as means ± SEM. *p≤0.05 versus WT, n = 3–4/group. (TIF) [file pone.0076553.s002.tif]

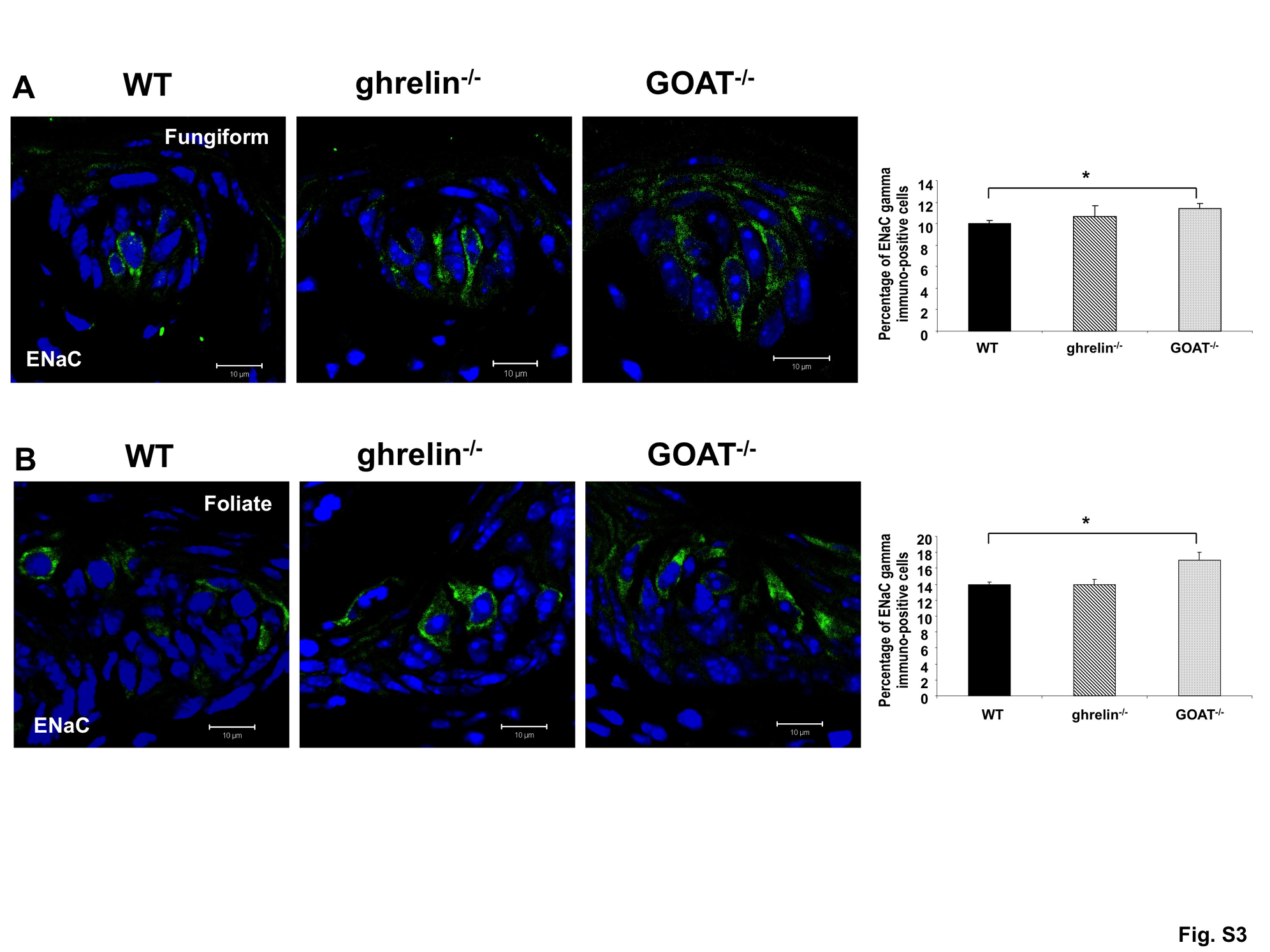

Supplement: Figure S3 — Expression of ENaC□in fungiform and foliate papillae of wild-type (WT), ghrelin−/− and GOAT−/− mice. (A), ENaC□ staining (green signals) in taste buds of fungiform papillae from wild-type (WT), ghrelin−/− and GOAT−/− mice. (B), ENaC□ staining (green signals) in taste buds of foliate papillae from wild-type (WT), ghrelin−/− and GOAT−/− mice. Blue = DAPI nuclear stain. The histograms associated with each taste bud figure represent the percentage of the immunopositive cells out of the total number of cells in each taste bud. Values are expressed as means ± SEM. *p≤0.05 versus WT, n = 4/group. Bars in each image are 10 µm. (TIF) [file pone.0076553.s003.tif]

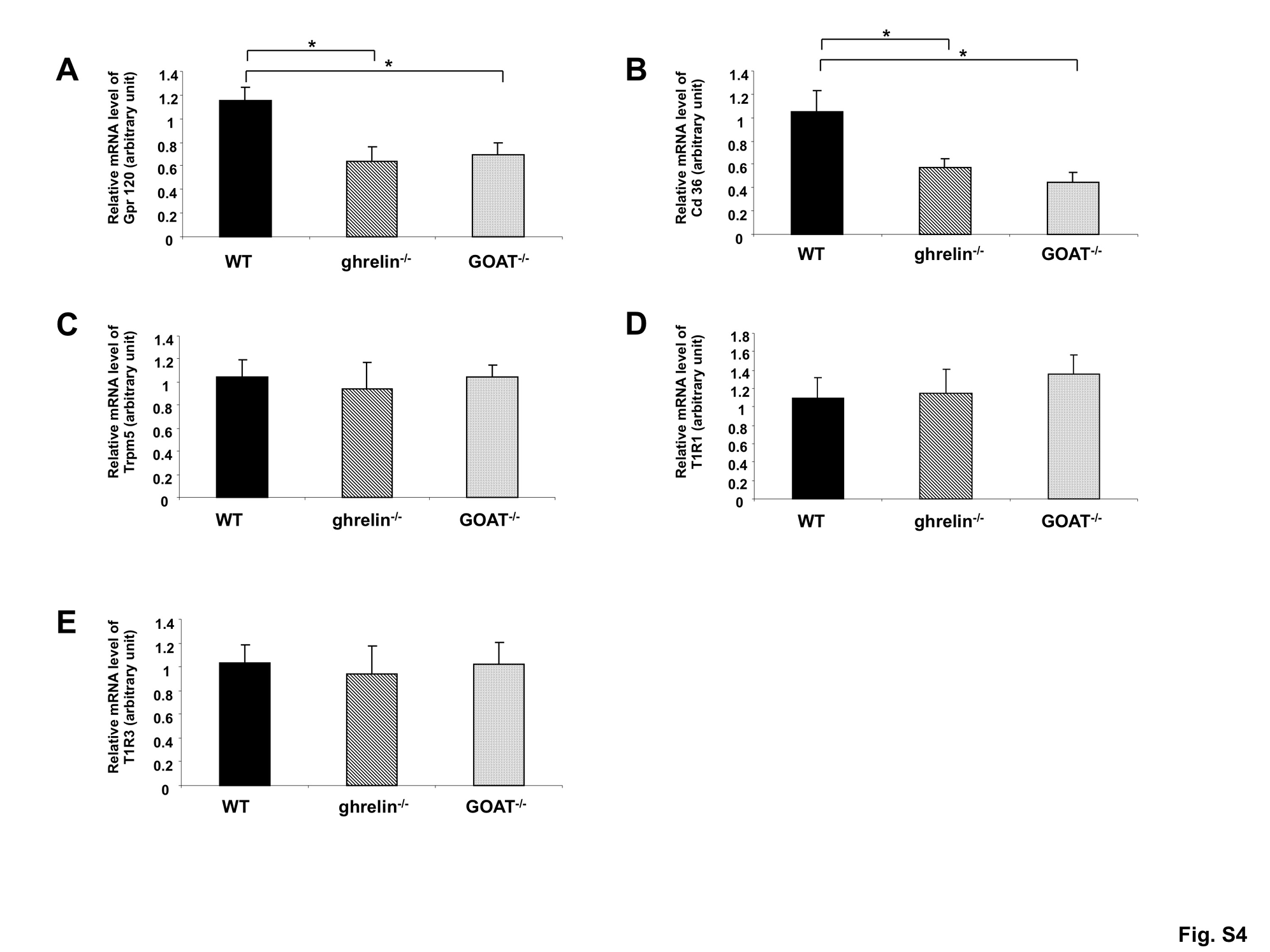

Supplement: Figure S4 — Real-time RT-PCR analysis. Relative mRNA expression of Gpr120 (A), Cd36 (B), Trpm5 (C), T1r1 (D) and T1r3 (E) was assessed by real-time RT-PCR in taste buds of wild-type (WT), ghrelin−/− and GOAT−/− mice. Values are expressed as means ± SEM. *p≤0.05 versus WT, n = 5/group. (TIF) [file pone.0076553.s004.tif]
